# Effects of Neuromuscular Electrical Stimulation of the Quadriceps and Diaphragm in Critically Ill Patients: A Pilot Study

**DOI:** 10.1155/2018/4298583

**Published:** 2018-07-08

**Authors:** Marcela Aparecida Leite, Erica Fernanda Osaku, Jaqueline Albert, Claudia Rejane Lima de Macedo Costa, Alessandra Madalena Garcia, Francieli do Nascimento Czapiesvski, Suely Mariko Ogasawara, Gladson Ricardo Flor Bertolini, Amaury Cezar Jorge, Péricles Almeida Delfino Duarte

**Affiliations:** ^1^Intensive Care Unit, Western Parana State University Hospital, Avenida Tancredo Neves 3224, Santo Onofre, 85806-470 Cascavel, PR, Brazil; ^2^Western Parana State University, Rua Universitária 2069, Jardim Universitário, 85819-110 Cascavel, PR, Brazil; ^3^Department of Medicine, Western Parana State University Hospital, Avenida Tancredo Neves 3224, Santo Onofre, 85806-470 Cascavel, PR, Brazil

## Abstract

**Background:**

Deep and respiratory muscle disorders are commonly observed in critically ill patients. Neuromuscular electrical stimulation (NMES) is an alternative to mobilize and to exercise that does not require active patient participation and can be used on bedridden patients.

**Objective:**

Evaluate the effectiveness of the NMES therapy in quadriceps versus diaphragm subjects in mechanical ventilation (MV).

**Methods:**

Sixty-seven subjects in MV were included, divided into 3 groups: (a) control group (CG, *n*=26), (b) stimulation of quadriceps (quadriceps group–QG, *n*=24), and (c) stimulation of diaphragm (diaphragm group–DG, *n*=17). The QG and DG patients received consecutive daily electrical stimulation sessions at specific points from the first day of randomization until ICU discharge. Respiratory and peripheral muscle strength, MV time, length of hospitalization, and functional independence score (the Functional Status Score-ICU) were recorded.

**Results:**

There were studied *n*=24 (QG), *n*=17 (DG), and *n*=26 (CG) patients. Peripheral muscle strength improved significantly in the QG (*p*=0.030). Functional independence at ICU discharge was significantly better in QG (*p*=0.013), and the QG presented a better Barthel Index compared to DG and CG (*p*=0.0049) and also presented better FSS compared to CG (*p*=0.001).

**Conclusions:**

Electrical stimulation of quadriceps had best outcomes for peripheral muscle strength compared with controls or electrical stimulation of diaphragm among mechanically ventilated critically ill subjects and promoted functional independence and decreased length of hospitalization.

## 1. Introduction

Limb and respiratory muscle disorders are commonly observed in critically ill patients [1]. Up to 25% of patients who require mechanical ventilation (MV) more than seven days in the intensive care unit (ICU) develop muscle weakness [2].

The neuromuscular disorders developed in the ICU comprises deep muscle weakness [3], including the respiratory muscles [4], loss of deep reflexes, and decrement of deep and superficial sensitivity [5]. This is associated with difficulty of weaning from MV, prolonged hospitalization, and increased mortality [6, 7]. Many risk factors are associated with the development of muscle weakness, including systemic inflammatory response, sepsis, severe organ dysfunction, hyperglycemia, prolonged immobility as well as the use of sedatives, neuromuscular blockers, and high doses of corticosteroids [8–10].

Early active mobilization in ICU patients is a safe and viable strategy to prevent the physical problems caused by immobility. However, patient cooperation may be necessary for a better outcome and adequate intervention [11, 12]. Unfortunately, not all critically ill patients can participate actively in early rehabilitation, often because of the use of sedatives or cognitive impairment [13, 14]. Therefore, in recent years, alternatives have been sought to help critical patients become more active, using passive mobilization strategies that include the neuromuscular electrical stimulation (NMES), an option that has been recently used for this purpose [15].

NMES is an alternative to mobilize and exercise because it does not require active patient participation and can be used on bedridden patients [16]. Deep muscle electrical stimulation has been shown to be beneficial for patients with muscle weakness developed in the ICU [17–20], with higher MRC scores in the electrical stimulation groups [21]. Most studies have shown that an electrical stimulation of deep muscle has beneficial effects [19, 20, 22], although no previous studies have shown whether training-specific respiratory muscles using an electrical stimulation can have overall benefits for ICU patients on MV. No previous studies have compared the application of an electrical stimulation in the quadriceps with transcutaneous stimulation in the diaphragm in critically ill patients. In addition, the few studies that addressed the electrical stimulation of the diaphragm specifically focused on COPD outpatients [23].

For this reason, the aim of this study was to evaluate, on a preliminary pilot study, the effectiveness of the NMES therapy in the muscle quadriceps or diaphragm of patients on MV.

## 2. Methods

### 2.1. Patients

This is a prospective, randomized pilot study that was conducted in accordance with the recommendations of Resolution 466/2012 of the Brazilian National Health Council. The project was approved by Western Paraná State University's Permanent Committee on Ethics and Research involving human beings, under protocol number 134.673. The participants, or their families, signed the informed consent term for participation in the study and use of data for publication. These data do not reveal the identity of the participants, and a “Term of Commitment for use of Data on File” was assigned by the authors, under recommendations of Resolution CNS 196/96 of Brazilian Ministry of Health, approved by the ethics committee.

The studied population consisted of the following: subjects admitted to the adult 14-bed (general, trauma, surgical, and medical) ICU of Western Parana State University Hospital, in Cascavel–Paraná–Brazil, between May and September of 2014. Inclusion criteria were age of 18 years or more and 24 hours of MV or more. Exclusion criteria were hemodynamic instability (although the use of vasoactive drugs is not a contraindication), pregnancy, body mass index (BMI) > 35 kg/m^2^, history of neuromuscular disease at admission, brain death, peripheral vascular diseases (arterial lower limb disease and deep venous thrombosis), bone fractures, use of an internal or external fixator, skin lesions, end-stage cancer, use of pacemakers, spinal injuries, or inability to receive an MRC score because of the cognitive state.

### 2.2. Study Design and Randomization

On the second day in ICU, the subjects were randomized into two groups: diaphragm group (DG) or quadriceps group (QG). The control group (CG) comprised subjects hospitalized in a time period immediately before the study began (January–April 2014), in which no electrical stimulation protocol had been established yet. DG and QG received consecutive daily sessions of electrical stimulation at specific points starting on the first day of randomization until the ICU discharge.

**Figure 1:**
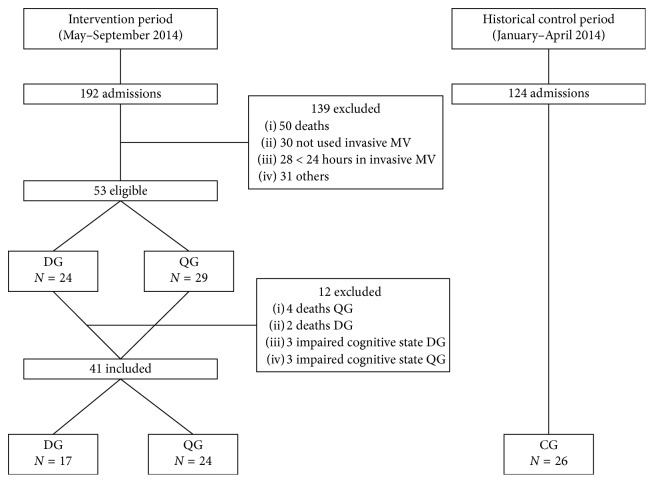
Flowchart of the patients admitted to the ICU and randomization process. MV, mechanical ventilation; CG, control group; DG, diaphragm group; QG, quadriceps group.

The study subjects were divided into three groups: (1) CG with 26 subjects who received regular treatment, that is, conventional physical therapy, which included gross motor therapy and respiratory therapy twice a day every day, including weekend, during their stay in the ICU; (2) DG composed of 17 subjects who received conventional physical therapy once a day, plus a daily session of electrical stimulation in the diaphragm; and (3) QG composed of 24 subjects who also received conventional physical therapy once a day, plus a daily session of electrical stimulation in the quadriceps (Figure 1).

QG and DG patients were randomized among the subjects who had been on MV for more than 24 hours, admitted to the ICU between May and September 2014, and who met the established inclusion criteria.

The CG group (historical) was chosen by convenience and included subjects who were admitted four months prior to the start of the protocol (January to April 2014). Therefore, they were not randomized. Their enrollment was based on the same inclusion and exclusion criteria.

### 2.3. Application of Neuromuscular Electrical Stimulation

NMES was performed using the Neurodyn Multicorrentes™ device (Ibramed, São Paulo, Brazil). The electric current parameters were different for each group.

**Figure 2:**
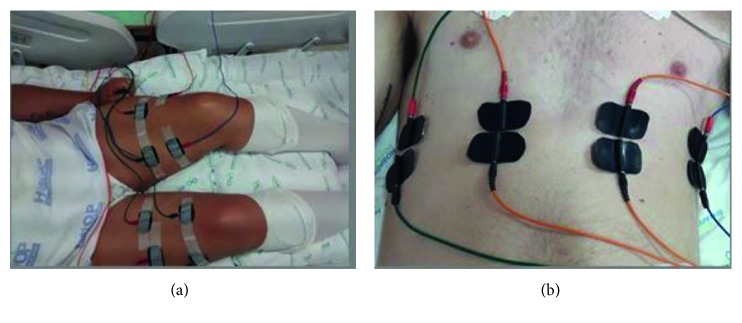
Neuromuscular electrical stimulation: (a) quadriceps and (b) diaphragm.

For the NMES of the quadriceps, the following parameters were used: Aussie current, synchronized impulse at a frequency of 50 Hz, 1 s pulse increase period, 8 s “on” (muscle contraction) period, 1 s pulse decrease period, and 30 s “off” (disconnection) period. After the skin was waxed and cleaned, a channel with two electrodes was applied to each vastus medialis, another channel with one electrode was applied to each vastus lateralis, and a third channel was applied to each rectus femoral (Figure 2(a)). Both limbs (quadriceps) were stimulated in each procedure. Although there were no previous studies of the application of this method in the ICU, an adaptation of the parameters was made based on previous experience and also on the use of the method in other populations [24].

The application of the NMES in the diaphragm was an adaptation of a previously established protocol [23, 25]: Aussie current, synchronized impulse at a frequency of 30 Hz, 1 s pulse increase period, 1 s “on” (muscle contraction) period, 1 s pulse decrease period, and 20 s “off” (disconnection) period; however, there was no synchronization between the contraction stimulus and breath of the patients. Two channels with two electrodes each were placed above and below the right and left sides of the xiphoid process within the seventh and eighth anterior intercostal space. In addition, two channels with two electrodes each were placed on the right and left midaxillary line of the seventh and eighth anterior intercostal space [23] (Figure 2). The intensity of contraction was assessed using the palpation of the muscle contraction of the diaphragm and visually.

Each session was performed for 45 min at intensities that produced visible contractions. In cases of doubt, contractions were confirmed through the palpation of the muscles involved.

The general clinical management, ventilatory strategy, weaning from mechanical ventilation, and tracheostomy were chosen and performed according to ICU protocols and routines and were applied indistinctly to the three groups.

### 2.4. Evaluation of Peripheral Muscle Strength and Respiratory Strength

Peripheral muscle strength was evaluated using the scale provided by the Medical Research Council (MRC). The movements were evaluated in three muscle groups in the upper limbs (shoulder abduction, elbow flexion, and wrist flexion) and lower limbs (hip flexion, knee extension, and ankle flexion) [26]. On this scale, the strength varies between 0 (plegic) and 60 (normal strength) points. A score below 48 indicates muscle weakness [27].

The MRC score was evaluated daily in each patient between the interruption of sedation and the discharge from ICU. On the moment of the evaluation, subjects were required to be at an adequate level of consciousness to respond to the following commands, adapted from De Jonghe et al. (2007): “open/close your eyes,” “look at me,” “show your tongue,” and “raise your eyebrows” [4]. The level of consciousness was evaluated using the Glasgow Coma Scale (GCS), and a score of 9 or above was required.

Respiratory muscle strength was evaluated using a manovacuometer (Gerar™–Class B, São Paulo) that measures the maximal inspiratory pressure (MIP).

Functional independence was evaluated using the Barthel Activities of Daily Living (ADL) Index and the Functional Status Score for the ICU (FSS-ICU). The Barthel ADL Index belongs to the field of evaluation of activities of daily living and measures functional independence associated with personal care, mobility, movement, and sphincter control (toilet use/bowels). In the original version, each item is scored according to the patient's performance with regard to tasks independently, with some help, or dependently. The overall score is calculated by attributing points in each category and depends on the time and assistance required for each subject. The score ranges from 0 to 100, at five-point intervals; higher scores reflect greater independence [28]. In this study, the Barthel ADL Index was applied once, at the time of ICU discharge.

FSS-ICU is a scale used in patient rehabilitation during hospitalization. It includes tasks that are more appropriate for critically ill subjects. The functional categories are scored between 1 (total assistance) and 7 (complete independence). A score of 0 is provided if the patient is unable to perform a task. FSS-ICU includes the following categories: rolling, supine to sitting transfers, unsupported sitting, sitting to stand transfers, and walking [13]. The evaluation with this scale was conducted on discharge from the ICU.

### 2.5. Statistical Analyses

The data collected were analyzed using the Statistica™ software version 7.0 (Stat Soft). The distribution pattern of the quantitative variables was evaluated using the Shapiro–Wilk test. The homogeneity of the variances was evaluated using the Levene test. The variables with normal and homogeneous distribution were compared among the three groups (QG, DG, and CG) using single-factor ANOVA. The variables analyzed over time were evaluated using ANOVA for repeated measures. These analyses were further evaluated using Fisher's LSD test.

For the other quantitative variables that did not fit the hypothesis of normality and homogeneity, the groups were compared using the Kruskal–Wallis nonparametric test. Dunn's test was applied to variables with statistical significance (*p* < 0.05).

All of the analyses were expressed as mean ± standard deviation and using box plots. The qualitative variables were compared among the groups using the chi-squared test of independence with Yates's correction. The level of significance was set at 5% for all cases (*p* < 0.05).

## 3. Results

During the five months of intervention study (groups QG and DG), a total of 192 subjects were hospitalized in the adult ICU. Of these, 53 were eligible for the study and 29 were randomized to the QG and 24 were randomized to the DG. Twelve patients were excluded: six subjects died and six did not have a level of consciousness to perform the MRC. Therefore, 41 subjects were evaluated (24 in the QG and 17 in the DG). To the CG, 26 subjects were recruited in the period of time before the NMES protocol was established (Figure 1).

The characteristics of the subjects of the three groups are presented in Table 1. There were no significant differences in age, sex, BMI, or APACHE II score among the 67 subjects included in the three groups. The cause of hospitalization was statistically significant among the three groups (*p*=0.042).

The duration of MV was significantly lower in the CG when compared with DG and QG (*p*=0.0001). However, there was no significant difference between DG and QG. The ICU length of stay was less in the QG, without significant difference between the groups. However, the patients of QG showed hospital length of stay significantly less when compared with the CG and DG (*p*=0.0031) (Table 2).

Regarding the predictive indexes MIP and RSBI, there was no significant difference between the analyzed groups. However, when compared with the last MIP, at the ICU discharge, with the first MIP, DG and QG presented higher respiratory muscle strength compared with CG (*p*=0.00003). Regarding MRC, all the groups presented significant improvement of muscular strength comparing the last and the first evaluation, but there was no significant difference between the groups. The QG presented a better Barthel Index compared with DG and CG (*p*=0.0049), also presented better FSS compared with CG (*p*=0.001), as did individual analysis of each item (Table 2).

As shown in Table 3, CG, QG, and DG were divided into two groups for comparisons: neurological subjects (traumatic brain injury and stroke) and nonneurological subjects (medical cases). The length of the sedation period did not differ significantly between these two groups.

The duration of MV was significantly shorter in the non-neurological QG patients compared with the nonneurological DG and CG subjects (*p*=0.028). The neurological subjects presented no statistically significant differences between the three groups in MV duration. No significant differences were observed in the predictive indices MIP and Rapid Shallow Breathing Index (RSBI) evaluated on the day of extubation (Table 3).

Peripheral muscle strength in the ICU discharge was significantly higher in the nonneurological QG subjects compared with the neurological subjects (*p*=0.030), and only the nonneurological QG subjects presented increase between their first and last evaluations (Table 3).

Regarding functional independence scores in ICU discharge, both the neurological and nonneurological subjects presented significant improvements in their Functional Status Scores in the ICU when an electrical stimulation was performed in the quadriceps (*p*=0.020 and *p*=0.013, resp.). Using the Barthel scale, a statistically significant difference was found only in the nonneurological group, with higher scores in the QG (*p*=0.035) (Table 3).

After ICU discharge, the nonneurological QG patients had significantly shorter hospital length of stay compared with the nonneurological DG and CG patients (*p*=0.004). The neurological subjects presented no statistically significant differences among the three groups (Table 3). During the study, there were no complications and problems related to the safety and use of NMES.

## 4. Discussion

In this study, daily consecutive electrical stimulation sessions produced better results in terms of MV duration, MIP, Barthel Index, and functional status than those in the control group. The NMES of the diaphragm did not present satisfactory results, compared with NMES of the quadriceps. This happened because electrical stimulation of the lower limbs promoted less hospitalization time and higher functional independence in critically ill subjects during discharge from ICU. Furthermore, electrical stimulation produced better results in nonneurological subjects. Peripheral muscle strength on the day of discharge from the ICU increased in all three groups; however, only the nonneurological QG subjects presented increase between their first and last evaluations.

The results of this study are consistent with a previous report [19], in that both groups presented higher MRC scores. However, the scores were significantly higher among electrically stimulated subjects compared with the control group (*p*=0.04), and subjects belonging to the electrical stimulation groups received daily sessions of simultaneous electrical stimulation in the vastus lateralis, vastus medialis, and peroneus longus from the second day after their admission into the ICU until their discharge. Gerovasili et al. [20] found a reduction in muscle mass loss after eight days of quadriceps and long fibular NMES, showing that NMES helps in the maintenance or gain of muscle strength.

Studies were performed on bedridden patients with COPD under long-term MV and the electrical stimulation of the quadriceps increased the muscle strength. These patients could move from a bed to a chair more quickly than subjects who received only active mobility therapy [16, 29]. In the present study, a significant and greater functional independence was found in the QG; this difference was obtained using the Barthel ADL Index and FSS-ICU at the time of discharge from the ICU.

Recent studies [23, 30] have reported good results about the increase in respiratory muscle strength with electrical stimulation of the diaphragm; however, the therapy was performed on nonhospitalized patients. In the present study, MIP was higher in the QG and DG when compared with the CG; this shows that electrical stimulation is a good factor to increase the respiratory muscle strength.

Electrical stimulation of the diaphragm did not produce good results, compared with those of electrical stimulation of the quadriceps. Until now, there have been no studies in relation to stimulation of the diaphragm in critically ill patients; the few studies that demonstrate the efficacy of electrical stimulation of the diaphragm were performed either on healthy patients in outpatient centers or in experiments on animal subjects [13, 25–31].

Due to the nature of the study, the efficacy of diaphragmatic stimulation was evaluated by clinical outcomes: effective weaning from MV and muscle strength presumed by the daily MIP measurement. However, there are other methods for evaluating diaphragm strength, through inspiratory muscle strength, such as sniff nasal inspiratory pressure (SNIP), that is, an alternative noninvasive test of inspiratory muscle function [32], the method of mechanomyogram (MMG) that measure the inspiratory strength using vibration sensors [33]. Therefore, the MIP is the most frequently utilized, noninvasive and direct method for measuring respiratory strength and is sensitive in detecting early respiratory muscle dysfunction [34, 35].

Another important point to be discussed is sepsis, which in the present study was not determined; however, studies performed with humans in ICUs and in animal models of critical subjects show that sepsis promotes muscle mass loss, decreased ability to generate force, loss of contractile proteins [36–38], and endothelial and microcirculation dysfunction [39, 40], affecting the diaphragm and limb muscles. During the inflammatory process, the endothelial regeneration capacity is impaired. According to Stefanou et al. [40], a single NMES session in the lower limbs seems to be beneficial, promoting increased counts of endothelial progenitor cells in critically ill patients with sepsis; however, these results should still be better determined in the diaphragm muscle, considering that in the present study, the electrical stimulation of the diaphragm may not have provided enough stimulation, and its effectiveness on the muscles in question present at deep locations may require the internal application of electrodes or different application parameters.

Electrical stimulation was well tolerated and, as previously reported [41], presented no side effects during the sessions. Because it does not require patient cooperation, electrical stimulation can be easily applied immediately after subject admission to the ICU. In the present study, electrical stimulation was immediately introduced after patient admission, after an average of 3.00 ± 2.09 days in the DG and 2.25 ± 2.21 days in the QG. This finding is similar to that in the study by Schweickert et al. [11], who showed a tendency toward improvement in functional independence among patients who received early rehabilitation, despite the different inclusion criteria and baseline data of the patients (in their study, rehabilitation began on an average of 1.5 days after patient admission into the ICU).

In addition to the issues considered above, this study has some limitations, some of which are intrinsic to its nature. This was a preliminary pilot study and further studies may be necessary, with amplification of the sample and variables analyzed. It was not a blind study, it was performed at a single site, the intervention period was short, and the sample was relatively small. Furthermore, the fact that most of the patients had neurological impairments may be a limiting factor in the analysis, particularly considering that muscle strength was evaluated and that an adequate level of consciousness was required. In addition, neurological patients themselves were grouped independently of their etiology and because of this the DG in nonneurological patients had only two subjects. Therefore, future analyses comparing subgroups (e.g., stroke versus trauma) may be necessary.

## 5. Conclusions

Electrical stimulation was beneficial for critically ill subjects by increasing peripheral muscle strength and functional independence and decreasing hospital length of stay.

There was a trend for better outcomes among subjects who received an electrical stimulation of the quadriceps, particularly among those without neurological impairment. This result indicates that the electrical stimulation of the quadriceps produces better outcomes compared with the electrical stimulation of the respiratory muscles.

The use of an electrical stimulation in the quadriceps is a promising technique in the rehabilitation of critically ill patients.

## Figures and Tables

**Table 1:** Baseline characteristics of the patients included in the study.

Variables	CG (*n*=26)	DG (*n*=17)	QG (*n*=24)	*p*
Age, years^*∗*^	42.4 ± 12.74	41.3 ± 24.26	48.8 ± 19.69	0.258
Male (%)	20 (76.9%)	15 (88.2%)	18 (75%)	0.606
BMI (kg/m^2^)^*∗*^	24.9 ± 2.85	27.5 ± 15.0	25.2 ± 3.03	0.572
APACHE II score (admission)^*∗*^	18.9 ± 1.93	17.9 ± 3.68	18.7 ± 4.01	0.616
Cause of hospitalization, *n* (%)				
Medical, nonneurological	1 (3.9%)	0	5 (20.8%)	0.042
Medical, neurological	9 (34.6%)	3 (17.6%)	4 (16.6%)
Traumatic brain injury	10 (38.5%)	12 (70.6%)	6 (25%)
Trauma, nonneurological	0	1 (5.9%)	5 (20.8%)
Postoperative emergency surgery	4 (15.4%)	0	2 (8.4%)
Postoperative elective surgery	2 (7.6%)	1 (5.9%)	2 (8.4%)
Comorbidities, *n* (%)				
Smoker	8 (30.7%)	5 (29.4%)	7 (29.1%)
Alcohol use	6 (23%)	4 (23.5%)	5 (20.8%)
COPD	8 (30.7%)	5 (29.4%)	7 (29.1%)	0.999
Asthma	3 (11.5%)	1 (5.8%)	2 (8.3%)
CHF	5 (19.2%)	3 (17.6%)	2 (8.3%)
Hypertension	8 (30.7%)	5 (29.4%)	7 (29.1%)
Diabetes mellitus	7 (26.9%)	2 (11.7%)	3 (12.5%)

APACHE, acute physiology and chronic health evaluation; BMI, body mass index; COPD, chronic obstructive pulmonary disease; CHF, congestive heart failure; CG, control group; DG, diaphragm group; QG, quadriceps group; SD, standard deviation. ^*∗*^Values are expressed as mean ± SD (*p* < 0.05).

**Table 2:** Comparison of the variables analyzed between groups.

Variables	CG (*n*=26)	DG (*n*=17)	QG (*n*=24)	*p*
Sedation (hours)	7.6 ± 4.64	11.5 ± 10.55	12.2 ± 11.25	0.3248
MV duration (hours)	15.8 ± 5.75^a^	27.5 ± 12.16^b^	23.3 ± 10.61^b^	**0.0001**
ICU length of stay (days)	12.8 ± 7.83	12.4 ± 4.45	9.7 ± 4.92	0.0998
Hospital length of stay (days)	25.4 ± 12.04^a^	29.3 ± 13.59^a^	18.2 ± 11.28^b^	**0.0031**
Minute volume (L/min)	9.8 ± 5.4	11.7 ± 5.2	10.3 ± 4.7	
Last minute volume (L/min)	11.9 ± 3.9	14.1 ± 6.9	12.8 ± 5.1	0.995
Minute volume (L/min), ext. day	13.1 ± 7.4	15.2 ± 7.2	13.2 ± 5.9	
First MIP (cmH_2_O)	−24.6 ± 14.13^a^	−23.2 ± 10.88^a^	−21.7 ± 11.68^a^	
Last MIP, ICU discharge (cmH_2_O)	−25.9 ± 9.59	−37.9 ± 10.31^b^	−40.4 ± 8.71^b^	**0.00003**
MIP, extubation day (cmH_2_O)	−25.9 ± 9.79	−29.1 ± 15.33	−30.8 ± 10.7	
First RSBI (rpm/l)	57.9 ± 40.42	43.2 ± 18.29	54.0 ± 34.15	
Last RSBI, ICU discharge (rpm/l)	52.1 ± 28.85	35.9 ± 14.17	48.9 ± 32.4	0.88708
RSBI (rpm/l), extubation day	53.6 ± 20.96	44.0 ± 19.45	56.9 ± 28.57	
First MRC	36.2 ± 5.55^a^	32.7 ± 7.22^a^	33.2 ± 8.47^a^	
				**0.00044**
Last MRC, ICU discharge	43.4 ± 6.45^b^	41.8 ± 11.14^b^	48.2 ± 11.48^b^	
Barthel	15.8 ± 14.04^a^	23.8 ± 24.97^a^	40.6 ± 30.08^b^	**0.0049**
FSS-ICU	14.6 ± 8.01^a^	21.5 ± 10.16^a^	29.1 ± 12.38^b^	**0.001**
In-bed transfers	2.2 ± 1.48^a^	3.2 ± 1.60	4.5 ± 2.00^b^	**0.0003**
Supine-to-sit transfer	2.0 ± 1.41^a^	3.1 ± 1.55	4.3 ± 1.86^b^	**0.0001**
Sitting at the bedside	2.4 ± 1.65^a^	3.5 ± 1.54	4.8 ± 1.83^b^	**0.001**
Unsupported sitting	3.6 ± 1.71^a^	4.8 ± 1.57	5.2 ± 1.75^b^	**0.0037**
Locomotion	1.1 ± 1.33^a^	2.3 ± 1.85	3.6 ± 2.33^b^	**0.0001**
GCS, ICU discharge	13.0 ± 1.64	12.7 ± 1.92	13.2 ± 1.87	0.5480

CG: control group; DG: diaphragm group; QG: quadriceps group; MV: mechanical ventilation; ICU: intensive care unit; Ext.: extubation; MIP: maximal inspiratory pressure; RSBI: Rapid Shallow Breathing Index; FSS-ICU: Functional Status Score for the ICU; MRC: Medical Research Council; GCS: Glasgow Coma Scale. Values are expressed as mean ± SD (*p* < 0.05). Different letters represent which group has statistical difference.

**Table 3:** Data of the groups and evolution according to causes of admission: neurological (medical, surgical, or trauma) and non-neurological.

	Neurological				Nonneurological			
	CG (*n*=19)	DG (*n*=15)	QG (*n*=10)	*p*	CG (*n*=7)	DG (*n*=2)	QG (*n*=14)	*p*
(i) Sedation (hours)	114.9 ± 115.51	95.8 ± 72.89	119.7 ± 121.52	0.866	79.5 ± 67.28	13.5 ± 19.09	54.5 ± 37.14	0.222
(ii) IMV duration (hours)	212.0 ± 151.95	207.2 ± 96.83	198.2 ± 124.96	0.963	161.4 ± 76.96	144.0 ± 24.04	104.2 ± 37.57	0.028
(iii) Minute volume (L/min), first	10.4 ± 5.1	11.9 ± 5.5	11.3 ± 5.7	0.598	8.3 ± 6.3	10.4 ± 2.9	9.7 ± 3.8	0.766
(iv) Minute volume (L/min), extubation day	13.5 ± 7.1	14.9 ± 7.7	15.1 ± 7.4	0.609	11.9 ± 8.6	17.5 ± 14.1	11.8 ± 4.3	0.444
(v) First MIP (cmH_2_O)	−24.2 ± 11.21	−24.0 ± 10.38	−24.5 ± 13.83	0.701	−25.7 ± 21.29	−17.5 ± 17.67	−20.0 ± 10.00	0.910
(vi) MIP, extubation day (cmH_2_O)	−25.5 ± 8.80	−28.3 ± 16.10	−33.0 ± 9.48	0.158	−27.1 ± 12.86	−35.0 ± 7.07	−29.2 ± 11.57	0.710
(vii) RSBI, extubation day (rpm/l)	54.7 ± 22.44	41.9 ± 19.48	51.5 ± 26.05	0.251	50.4 ± 17.40	60.0 ± 2.72	60.7 ± 30.59	0.700
(viii) Barthel	15.7 ± 14.16	24.3 ± 25.55	32.5 ± 32.42	0.185	15.7 ± 14.84	20.0 ± 28.28	46.4 ± 28.04	0.035
(ix) FSS-ICU	14.0 ± 6.87	21.4 ± 9.47	23.8 ± 13.53	0.020	16.2 ± 11.01	23.0 ± 19.79	33.0 ± 10.33	0.013
In-bed transfers	2.3 ± 1.56	3.2 ± 1.42	3.5 ± 1.84	0.115	2.1 ± 1.34	3.5 ± 3.53	5.2 ± 1.84	0.006
Supine-to-sit transfer	2.0 ± 1.31	3.2 ± 1.47	3.5 ± 1.90	0.028	2.1 ± 1.77	3.0 ± 2.82	5.0 ± 1.61	0.006
Sitting at the bedside	2.4 ± 1.54	3.6 ± 1.44	4.0 ± 1.88	0.028	2.4 ± 2.07	3.0 ± 2.82	5.4 ± 1.60	0.005
Unsupported sitting	3.7 ± 1.65	4.9 ± 1.57	4.4 ± 2.06	0.170	3.2 ± 1.97	4.5 ± 2.12	5.9 ± 1.20	0.004
Locomotion	1.0 ± 1.20	2.2 ± 1.79	2.7 ± 2.40	0.027	1.4 ± 1.71	3.0 ± 2.82	4.3 ± 2.09	0.019
(x) GCS at ICU discharge	13.0 ± 1.59	12.8 ± 1.82	12.3 ± 1.94	0.596	13.2 ± 1.88	12.5 ± 3.53	14.0 ± 1.51	0.451
(xi) First MRC	37.5 ± 4.64	33.9 ± 5.16	33.0 ± 10.67	0.070	32.5 ± 6.50	24.0 ± 16.97	33.4 ± 6.93	0.689
(xii) Last MRC	44.1 ± 5.74	43.8 ± 8.62	43.4 ± 12.58	0.745	41.7 ± 8.30	27.0 ± 21.21	51.7 ± 9.63	0.030
(xiii) Hospital length of stay (days)	24.5 ± 10.10	27.2 ± 12.94	22.7 ± 13.21	0.633	27.8 ± 16.99	45.0 ± 7.07	15.0 ± 8.84	0.004

IMV: invasive mechanical ventilation; MIP: maximal inspiratory pressure; RSBI: Rapid Shallow Breathing Index; FSS-ICU: Functional Status Score for the ICU; GCS: Glasgow Coma Scale; MRC: Medical Research Council; CG, control group; DG, diaphragm group; QG, quadriceps group. Values are expressed as mean ± SD (*p* < 0.05).
